# Stereotactic body radiotherapy to the left lung in right lateral decubitus: A challenging case report

**DOI:** 10.1002/acm2.70564

**Published:** 2026-04-23

**Authors:** Jochen Cammin, Elizabeth Manuel, Shifeng Chen, Zaker Rana, Matthew J. Ferris

**Affiliations:** ^1^ Department of Radiation Oncology University of Maryland School of Medicine Baltimore Maryland USA; ^2^ Department of Radiation Oncology University of Maryland Medical Center Baltimore Maryland USA

**Keywords:** 4D‐CT, limited‐arc CBCT, lung SBRT, off‐center isocenter, PSQA

## Abstract

This case report describes a lung stereotactic body radiotherapy (SBRT) treatment complicated by the patient's inability to tolerate standard supine positioning, requiring simulation and delivery in the right lateral decubitus position. Five major workflow challenges were encountered involving patient positioning, 4DCT acquisition, treatment planning involving gantry‑clearance limitations, patient‑specific QA, and CBCT‑based image guidance. Customized solutions, including non‐standard respiratory‐surrogate placement for 4DCT, off‑center isocentering, customized limited‑arc CBCT, and measurement‑based patient‐specific QA, enabled safe and effective treatment. This case highlights the need for adaptable SBRT workflows for patients unable to tolerate conventional positioning.

## 1 INTRODUCTION

Stereotactic body radiotherapy (SBRT) is a widely used technique for treating early‐stage or oligometastatic lung cancer. This highly focused treatment delivers large doses per fraction with steep dose gradients, requiring accurate, stable, and reproducible patient positioning. Pre‐treatment imaging with high‐quality cone‐beam CT (CBCT) is considered standard‐of‐care. We present a lung SBRT case that posed several unique challenges at multiple steps across simulation, planning, and image‐guided radiotherapy and required deviations from our standard procedures.

## 2 CASE PRESENTATION

A 68‐year‐old female former smoker with an 80–100 pack‐year history was referred to our department with a diagnosis of non‐small lung cancer in the left lung, upper lobe, stage IA2 (cT1lb, cN0, cM0). The peripheral nodule measured approximately 1.6 cm × 1.3 cm on the most recent radiological exam, an interval volume increase by 28% from an exam performed 5 months earlier. The patient was not a surgical candidate due to performance status and lung function. After consultation with the radiation oncologist, the patient consented to lung SBRT, prescribed (Rx) to 48 Gy in 4 fractions. This case presented five challenges related to the patient's performance status, each requiring deviations from our standard lung SBRT workflows.

### 2.1 Challenge 1: Tolerable treatment position

**FIGURE 1:**
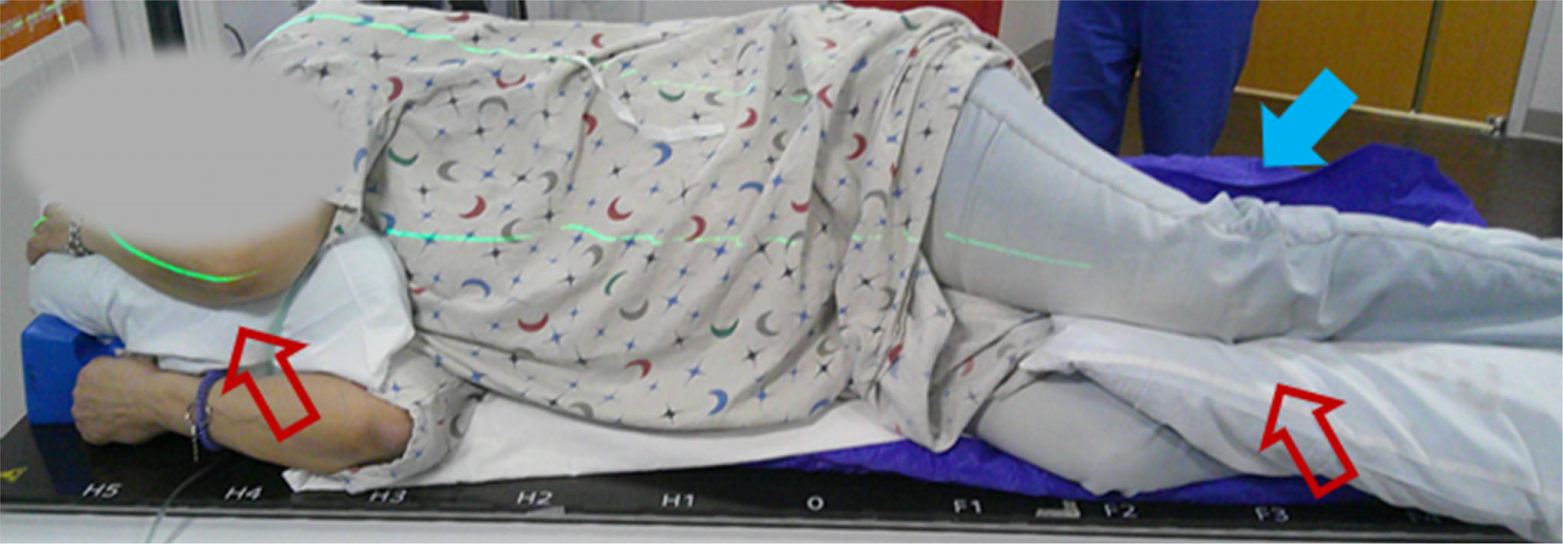
Patient setup and immobilization in the lateral decubitus position during CT simulation. The blue filled arrow indicates the immobilization device built up posteriorly to reduce rotational uncertainties. The red open arrows indicate pillows between arms and legs to promote comfort and reproducibility.

**FIGURE 2:**
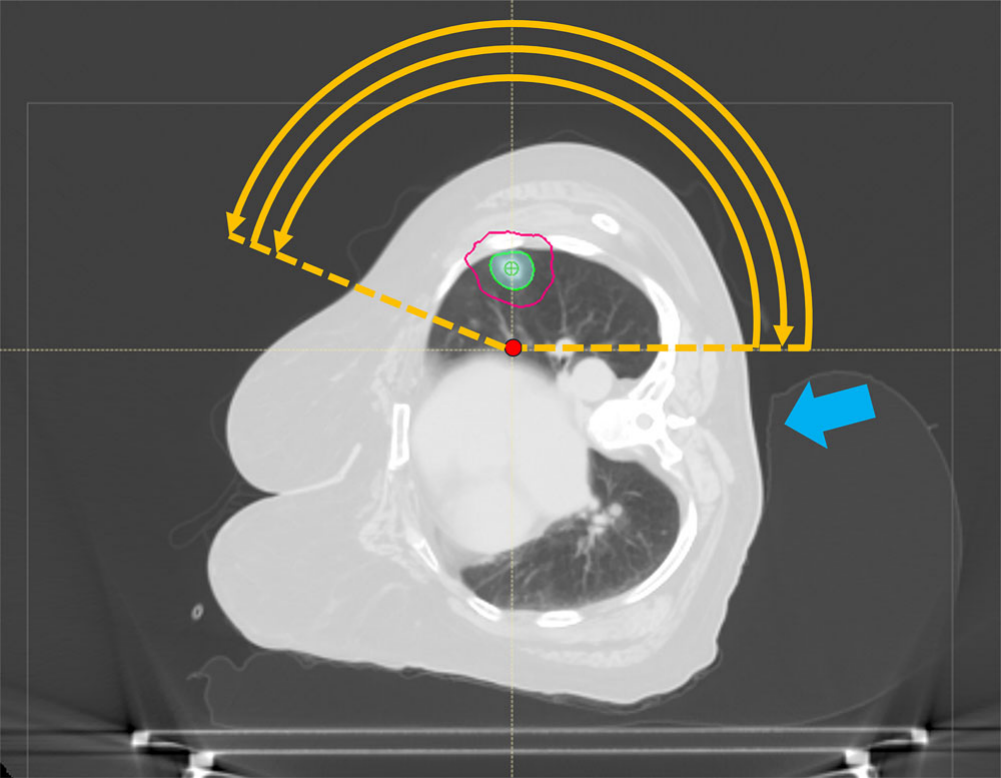
Planning CT dataset. The red dot indicates the shifted isocenter location. The shaded blue structure indicates the PTV, the 100% isodose line is shown in green and the 50% isodose line is shown in magenta. The orange arcs indicate the beam trajectories. The blue filled arrow indicates the immobilization device built‐up posteriorly.

Standard practice in our clinic includes CT simulation with the patient supine, arms above the head on a wing board, an upper Vac‐Lok bag for immobilization of the head, neck, and thorax, a lower Vac‐Lok bag for immobilization of the legs, and a knee sponge for comfort. However, this patient required chronic supplemental oxygen, particularly, when lying flat, and could not tolerate the supine position. Elevation using a breast board did not resolve the patient's respiratory difficulty. A prone position was discussed but not attempted. Ultimately, the patient was immobilized and simulated in the right lateral decubitus position, which was the patient's normal sleeping position and the only position the patient could tolerate. Rotational stability and reproducibility were facilitated by using a longer Vac‐Lok bag that reached from the shoulder to the calves and by building up the bag on the patient's posterior side (blue filled arrows in Figures 1 and 2). Pillows were placed between the arms and legs to improve patient comfort and stability (red open arrows in Figure 1).

### 2.2 Challenge 2: Respiratory‐correlated 4DCT

**FIGURE 3:**
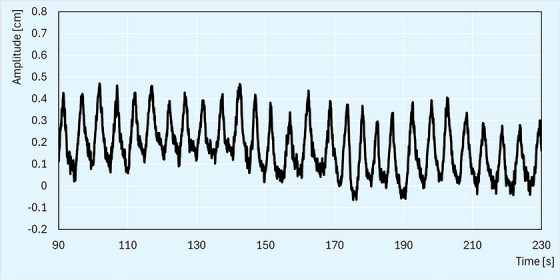
Respiratory trace of the patient's breathing during 4DCT simulation.

A free‐breathing simulation CT was acquired, including a fast 3D scan followed by a respiratory‐correlated 4DCT to assess tumor motion. Our 4DCTs are acquired with the RGSC system (Varian Medical Systems, Palo Alto, CA), which uses a reflector block tracked by a wall‐mounted camera. Manufacturer guidelines recommend placing the reflector block at midline on the sternum or upper abdomen in the supine position. This was not feasible in the lateral decubitus position. Instead, the reflector block was placed on the patient's left flank. The breathing‐induced surface motion at that location measured approximately 0.4 cm, which was above the 0.2 cm minimum threshold required for the system to properly capture motion information. The patient's breathing was fairly regular (Figure 3), and the 4DCT was successfully acquired without the need for respiratory coaching. Tumor motion assessed on the phase‐based motion bins was approximately 0.8 cm in the cranio‐caudal direction. No further motion‐mitigation strategies, such as breath‐hold techniques, were attempted due to the patient's respiratory limitations.

### 2.3 Challenge 3: Treatment planning and gantry clearance

**FIGURE 4:**
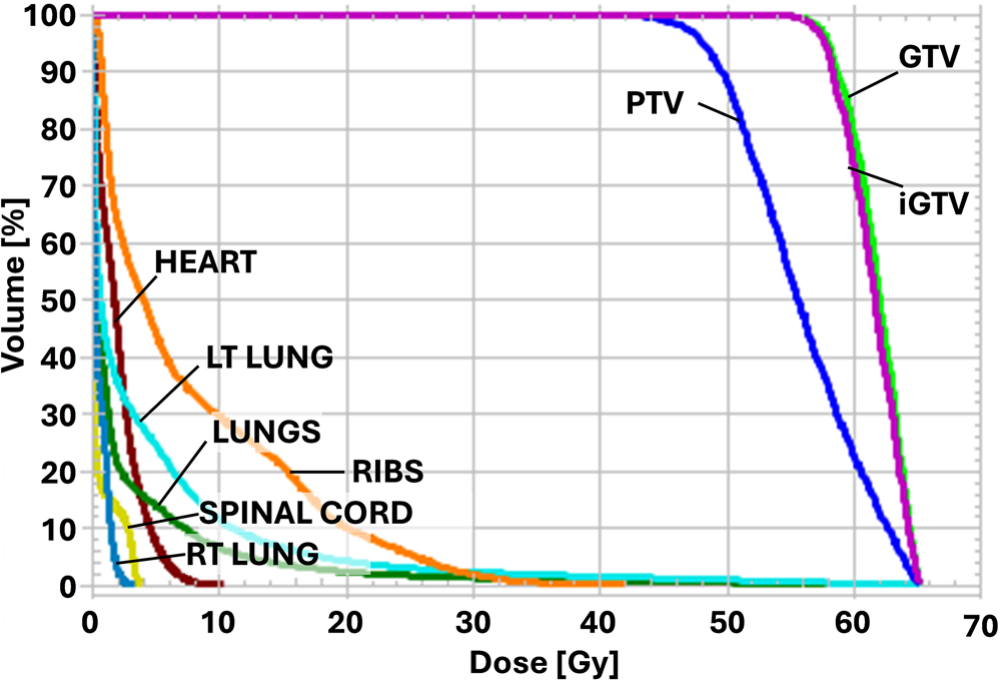
Dose‐volume histograms for the targets (GTV, iGTV, PTV) and select OARs.

Treatment planning followed standard institutional practice for lung SBRT. The motion‐average dataset derived from the 4DCT served as the primary planning dataset. An ITV encompassing the GTV in all 10 motion bins, and a PTV was created with a uniform 0.5 cm margin. The GTV volume was 3.8 cm^3^ and the PTV volume was 15.3 cm^3^. Because the tumor was in the left lung and the patient would be treated in the right decubitus position, placing the beam isocenter at the geometric center of the PTV would have required lowering the couch about 30 cm below the linac isocenter. To reduce gantry‐clearance risks, the isocenter was shifted 5 cm medially. Three partial treatment arcs were designed with trajectories from 91° (gantry near horizontal at the patient's posterior side) to 291° (21° above horizontal on the patient's anterior side), alternating counter‐clockwise and clockwise rotations (Figure 2). The 291° stop angle avoided beams going through the breast tissue, whose position might be unreproducible from one fraction to the next, and which may also not be visualized in the setup CBCT images. Beam apertures and monitor units were optimized in the RayStation treatment‐planning system (TPS), (v11B, RaySearch Laboratories, Stockholm, Sweden) using volumetric‐modulated arc therapy (VMAT) and 6FFF photon beams. A high‐quality SBRT plan was achieved that met RTOG 0915 guidelines^1^ for the target and critical organs‐at‐risk (OARs), except for the maximum point dose to the ribs (Figure 4): 95.5% of the PTV was covered by 100%Rx, 99.6% of the PTV was covered by 90%Rx with a dose maximum of 136%Rx. The conformity index was 1.00 (ratio of prescription isodose to PTV volume), the gradient index was 4.4 (ratio of 50% isodose volume to target volume), the D2cm was 45%Rx (dose to normal tissue located 2 cm or more from the PTV), the volume of tissue outside the PTV receiving > 105%Rx was 0.3 cc (0.05% of the PTV volume), the spinal cord maximum point dose was 3.9 Gy, and the lung V20Gy was 2.2%. The V32Gy to the ribs was 0.9 cc, but the maximum point dose was 42.1 Gy due to < 1 mm proximity to the PTV (RTOG 0915 guideline: ≤40 Gy).

### 2.4 Challenge 4: Patient‐specific quality assurance

At our institution, patient‐specific QA (PSQA) for VMAT plans is primarily calculation‐based, using Mobius3D (v4.0.1, Varian Medical Systems, Palo Alto, CA) as an independent dose‐calculation engine. For this plan, Mobius3D showed several discrepancies that exceeded institutional tolerances and recommendations in AAPM Task Group Report 218^2^ including target mean‐dose and D95% coverage differences greater than 5% and very low gamma‐passing rates (GPR^3^) of less than 1% for the GTV and 15% for the PTV using a 3%/2 mm criterion with 10% dose threshold and global normalization in absolute dose. These deviations were attributed to differences in homogeneity corrections and MLC modeling between the clinical TPS and the secondary dose calculation, potentially exacerbated by the off‐center isocenter (see discussion section). Following institutional policies, measurement‐based QA was performed instead using a 2D diode array (SRS MapCheck, Sun Nuclear, Melbourne, FL). The detector array (77 × 77 mm^2^ size, 2.47 mm detector spacing) was positioned horizontally on the patient table at isocenter and then shifted up by 5 cm to reflect the shifted treatment isocenter. The unmodified VMAT plan was delivered to the device and resulted in a 98.6% GPR (using global normalization in absolute dose, 3%/2 mm, 10% threshold), which met TG 218 tolerances.

### 2.5 Challenge 5: Image‐guided RT (IGRT) with low couch position

**FIGURE 5:**
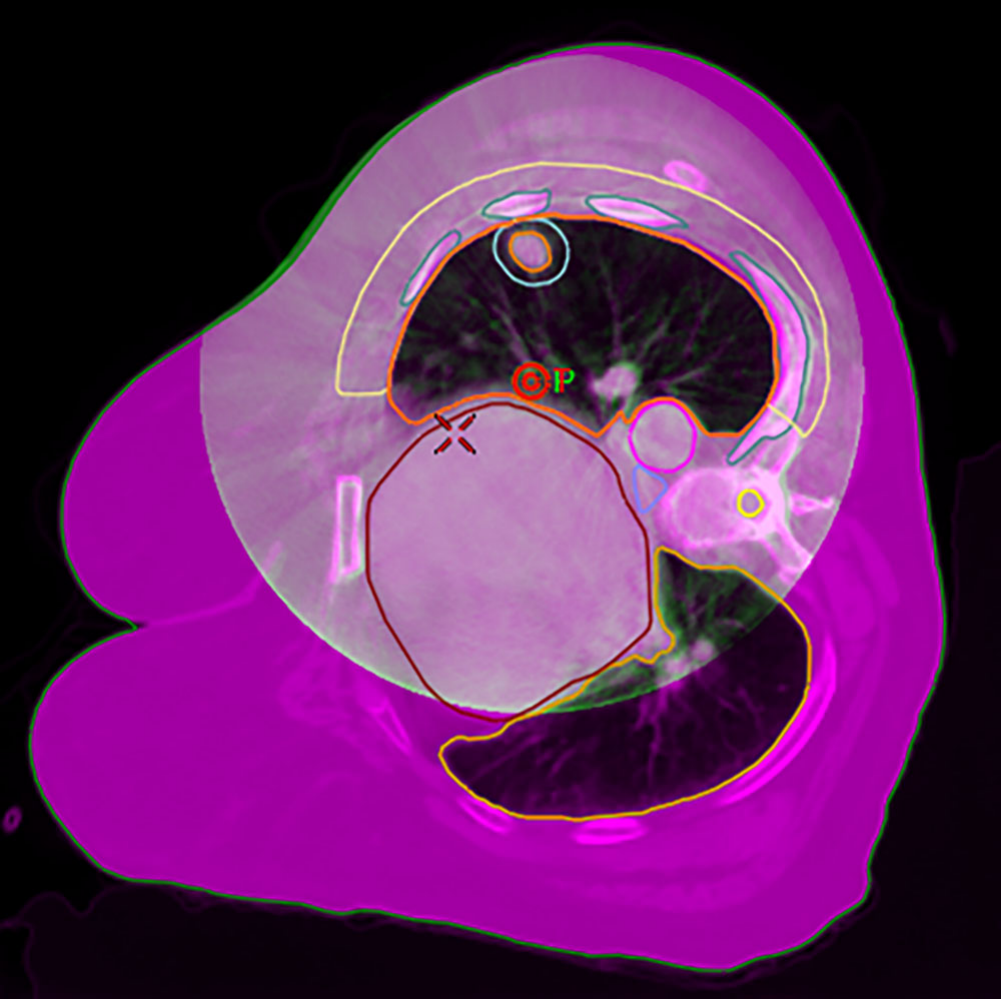
Second fraction setup CBCT (green/white) overlaid over the planning CT dataset (magenta).

Although gantry‐clearance issues during treatment delivery were mitigated by keeping the gantry in the upper hemisphere, our lung SBRT protocol required IGRT with orthogonal radiographs followed by CBCT for alignment. The standard thorax CBCT mode on Varian TrueBeam linacs uses a half‐bowtie filter and full gantry rotation to reconstruct images with a 46 cm axial field‐of‐view (FOV). Even with the shifted isocenter, there were concerns about couch‐gantry clearance with the gantry head in the lower hemisphere. The automatic couch‐centering feature on the Varian TrueBeam linac could not be used as it would place the couch too high, leading to collisions between the gantry head and the patient's left shoulder. To accommodate these limitations, a custom CBCT mode was created using the full‐bowtie filter and a limited 200° trajectory. The gantry start/stop angles were set to 100° and 260°, respectively, keeping the gantry in the upper hemisphere. This custom mode provided a 26 cm FOV, which was adequate for visualizing the tumor and surrounding anatomy but excluded the patient's right side and breast tissue (Figure 5).

## 3 DISCUSSION

Treatment setup, imaging, and treatment deliveries were ultimately uneventful. The need for minor roll adjustments during patient alignment to the room lasers and the patient's anxiety contributed to slightly longer pre‐imaging setup times, but quantitative timing data for this portion of the workflow was not obtained. The average time from start of CBCT acquisition to completion of treatment delivery was 7 min 39 s (range 5:23 to 10:59), which is comparable to treatment times for patients treated in the supine position. After imaging, translational shifts larger than 1 cm and rotational adjustments larger than 2 degrees were necessary for three of the four fractions (Table 1). Six‐degree‐of‐freedom (6DOF) couch corrections allowed these adjustments without the therapists having to re‐enter the treatment room. The roll required the largest correction, with values close to the couch's 3° physical limit for the first two fractions. This aligns with previous reports describing similar challenges when treating patients in the lateral decubitus position.^4^ The need to deviate from our standard PSQA workflows and acquire measurement‐based dose‐verification due to the shifted isocenter added additional time to chart and QA reviews. Discrepancies between TPS and Mobius3D for off‐center targets, similar to the ones observed in this case, have been reported previously.^5^

**TABLE 1:** Table shifts and rotations applied during IGRT patient setup for the four treatment fractions.

Fraction	Vertical (cm)	Longitudinal (cm)	Lateral (cm)	Pitch (deg)	Roll (deg)	Rotation (deg)
1	−1.4	0.5	1.7	1.1	2.9	2.5
2	0.7	−1.3	−0.8	−0.9	2.8	−1.0
3	0.8	−1.4	−0.4	0.3	2.5	−1.4
4	0.7	−0.7	0.6	0.2	0.6	−1.4

Additional considerations and educational discussion points:

1. *Verification imaging after couch adjustments*: Additional CBCT imaging following the initial 6DOF couch corrections may be warranted to confirm that the patient has not unintentionally shifted position in response to the pitch and roll adjustments.
2. *Alternative planning and delivery strategies*: A static‐gantry IMRT plan may be considered as an alternative to VMAT to avoid gantry angles that would result in clearance issues. When beams in the lower gantry hemisphere are used, a temporary lateral couch shift may provide sufficient clearance for gantry rotation. In such scenarios, additional imaging may be necessary to verify accurate patient position before the treatment proceeds.
3. *Alternative motion‐mitigation strategies*: A free‐breathing treatment was used due to this patient's respiratory performance status. For patients who require lateral‐decubitus positioning for other reasons, deep‐inhalation breath hold or respiratory‐gated treatments may provide additional motion control. However, these approaches generally prolong both simulation and treatment times, and it must be verified that any respiratory‐surrogate device used for monitoring provides a reliable and reproducible signal when the patient is treated in the lateral‐decubitus position.
4. *Discrepancy between TPS and secondary dose calculation*: To investigate potential sources for dosimetric differences between TPS and secondary dose calculation, a stepwise evaluation was performed. First, all tissues within the body contour were overwritten with water density in the TPS, and the clinical plan was recalculated. GPRs and dose differences improved, indicating that homogeneity corrections contributed to some of the discrepancies (Table 2, “Plan A [water density]”). However, the GTV and PTV GPRs remained low (9.4% and 50.0%, respectively). Next, a modified plan was created with the isocenter placed at the GTV's center‐of‐mass. After reoptimization to match the clinical plan and meet all RTOG 0915 criteria, *D*_mean_ and D95% differences improved to < 5% (Table 2, “Plan B [CT density]”). GPRs increased from 0.8% to 62% for the GTV and from 14.5% to 68.2% for the PTV. The global GPR was 95.8% and met TG 218 tolerances. These improvements suggest that reducing off‐axis beam contributions minimized discrepancies, implicating that Mobius3D MLC modeling for obliques rays as a likely source of the remaining differences. Finally, recalculating the modified plan with the body overwritten to water met all tolerance criteria, with GPR > 95% for all structures and *D*_mean_ and D95% differences < 5% (Table 2, “Plan B [water density]”).

**TABLE 2:** Comparison of Gamma‐passing rates (GPR) and relative dosimetric differences between the dose distribution from RayStation and Mobius3D.

	GPR			*D*_mean_		D95%	
	WHOLE BODY	GTV	PTV	GTV	PTV	GTV	PTV
Plan A [CT density]	92.7%	0.8%	14.5%	−6.4%	−7.9%	−7.5%	−9.6%
Plan A [water density]	95.3%	9.4%	50.0%	−4.3%	−5.4%	−5.2%	−5.5%
Plan B [CT density]	95.8%	62.0%	68.2%	−3.5%	−4.7%	−4.5%	−4.8%
Plan B [water density]	97.4%	100%	96.2%	−1.6%	−2.2%	−1.1%	−0.8%

*Note*: Plan A is the clinical plan with a shifted isocenter. Plan B is an alternate plan with the isocenter placed inside the GTV. 3%/2 mm with a 10% threshold and absolute dose was applied for the GPR calculation. *D*_mean_ is the mean dose and D95% is the dose to 95% of the structure volume.

## 4 CONCLUSION

Despite multiple challenges in simulation, planning, PSQA, and IGRT, careful evaluation of workflow limitations and corresponding customizations enabled safe and effective SBRT delivery in this complex patient scenario.

## AUTHOR CONTRIBUTIONS

**Jochen Cammin**: conceptualization; analysis; original draft preparation. **Elizabeth Manuel**: treatment planning; manuscript review & editing. **Shifeng Chen**: conceptualization; supervision; manuscript review & editing. **Zaker Rana**: project administration; manuscript review. **Matthew J. Ferris**: supervision; manuscript review & editing.

## CONFLICT OF INTEREST STATEMENT

JC is a member of the JACMP Board of Associate Editors. SC is a member of the AAPM Board of Directors. All other authors have no relevant conflicts of interest to disclose.

## DECLARATION OF GENERATIVE AI AND AI‐ASSISTED TECHNOLOGIES IN THE WRITING PROCESS

During the preparation of this work, the authors used ChatGPT (v5) in order to assist with improving grammar and language. After using this tool, the authors reviewed and edited the content as needed and take full responsibility for the content of the published article.

## Data Availability

Research data are not available for this case.
